# Physical risk factors influencing wheeled mobility in children with cerebral palsy: a cross-sectional study

**DOI:** 10.1186/s12887-016-0707-6

**Published:** 2016-10-10

**Authors:** Elisabet Rodby-Bousquet, Ginny Paleg, Jackie Casey, Alicja Wizert, Roslyn Livingstone

**Affiliations:** 1 Centre for Clinical Research, Uppsala University, Vestmanland County Hospital, SE-721 89 Västerås, Sweden; 2 Department of Clinical Sciences Lund, Orthopaedics, Lund University, University Hospital, Lund, Sweden; 3 Montgomery County Infants & Toddlers Program, Maryland, USA; 4 Institute of Nursing and Health Research, School of Health Sciences, University of Ulster, Coleraine, Northern Ireland; 5 Sunny Hill Health Centre for Children, Vancouver, British Columbia Canada

**Keywords:** Cerebral palsy, Children, Wheelchairs, Mobility, Power, Hand function, Range of motion, GMFCS, MACS

## Abstract

**Background:**

There is a lack of understanding of the factors that influence independent mobility and participation in meaningful activities. The purpose of this study was to analyse physical factors influencing independent use of manual and power wheelchairs in a total population of children with cerebral palsy (CP).

**Methods:**

A cross-sectional study based on the most recent examination of all children with CP, born 2002–2013, reported into the Swedish cerebral palsy registry (CPUP), from January 2012 to June 2014. There were 2328 children (58 % boys, 42 % girls), aged 0–11 years, at all levels of gross motor function and hand function. Hazard ratios adjusted for age and sex were used to calculate the risk for not being able to self-propel based on Gross Motor Function Classification System (GMFCS) levels, upper extremity range of motion and hand function including Manual Ability Classification System (MACS), House functional classification system, Thumb-in-palm deformity, Zancolli (spasticity of wrist/finger flexors) and bimanual ability.

**Results:**

In total 858 children used wheelchairs outdoors (692 manual, 20 power, 146 both). Only 10 % of the 838 children self-propelled manual wheelchairs, while 90 % were pushed. In contrast 75 % of the 166 children who used power mobility outdoors were independent. Poor hand function was the greatest risk factor for being unable to self-propel a manual wheelchair, while classification as GMFCS V or MACS IV-V were the greatest risk factors for not being able to use a power wheelchair independently.

**Conclusions:**

The majority of children with CP, aged 0–11 years did not self-propel manual wheelchairs regardless of age, gross motor function, range of motion or manual abilities. Power mobility should be considered at earlier ages to promote independent mobility for all children with CP who require a wheelchair especially outdoors.

## Background

There is a relationship between independent mobility, overall development and participation for children with disabilities [1]. Infants and toddlers with motor impairments, and/or intellectual impairment, benefit from independent mobility experience to enhance overall development [2], while older children and adolescents need efficient mobility in order to enhance participation at home, at school and in the community [1–3].

The Gross Motor Function Classification System (GMFCS) [4, 5] classifies children with cerebral palsy (CP) according to their level of gross motor function and, in particular, method of locomotion. For example, children classified as GMFCS III or IV may be able to walk with aids but often use wheeled mobility, especially outdoors and in the community, whereas self-mobility is severely restricted for those children classified as GMFCS V. Many factors that are not well understood appear to limit independent wheeled mobility use in children with CP [6, 7].

GMFCS level and age do not necessarily predict children’s activity and participation as there is an interaction between primary impairments (e.g. spasticity), secondary impairments (e.g. range of motion) and environmental factors such as family support or community services [8]. Even within GMFCS levels, there is considerable variability in the mobility methods that children use across settings as personal and contextual factors influence level of independence [9].

Ability to use the hands functionally does not necessarily correlate with gross motor function abilities. The Manual Ability Classification System (MACS) classifies how children with CP handle objects in daily life [10]. For example, children classified as MACS level I can handle most objects easily while children classified as level V do not handle objects and may, at best, be able to activate a single switch.

A large study, of 519 Canadian children with CP aged 4–12, revealed that only 20 of the 302 children (7 %) in GMFCS III-V were able to self-propel manual wheelchairs. While 37 children (12 %) used power mobility, and 202 (67 %) were passively pushed at school or outdoors in the community [7]. Given that children with significant motor and cognitive impairments have been shown to use power mobility competently [11], the authors speculate that environmental factors such as funding restrictions, may have contributed to these low numbers. In Sweden, a study of 562 children with CP aged 3–18 years [6] revealed similar results, where 228 used wheelchairs outdoors and only 66 (29 %) were independent (18 using manual, 36 power and 12 both), while 162 (71 %) were passively pushed in manual wheelchairs. Only five children within the total study used power mobility prior to school age.

The literature lacks clarity about factors influencing use of different mobility aids by children with CP. Attitudes, personal factors and environmental considerations have been suggested as influencing factors [6, 7, 9]. Prior studies [6, 7] suggest that even children classified as GMFCS II-III are not independent in self-propelling manual wheelchairs. The number of children unable to self-propel manual wheelchairs [6] correlates with those classified as having poor hand function in a similar population [12], suggesting that arm and hand function may be relevant factors to explore further.

The purpose of this cross-sectional study was to analyse data from a total population of children with CP and to explore relationships between different physical factors influencing independent use of wheeled mobility. Our hypothesis was that hand function and upper extremity range of motion would have an influence on independent wheeled mobility use.

## Methods

In Sweden all children with suspected CP are included in the national follow-up programme and quality registry known as CPUP [13, 14]. Inclusion and exclusion criteria are defined by the Surveillance of Cerebral Palsy in Europe [15]. The diagnosis is verified by a Neuropaediatrician at the age of 4 to 5 years. The data represents all regions of Sweden and the vast majority of families of children with CP (>95 %) agree to participate [16]. Data were analysed on power and manual wheelchair use indoors and outdoors in relation to age, sex, GMFCS [4, 5] and MACS levels [10]. Influence on independent mobility of other factors such as upper extremity range of motion, bimanual ability, thumb-in-palm and ability to actively extend wrist and fingers was also explored. Ethical approval was granted by the Medical Research Ethics Committee at Lund University, and permission obtained to extract data from the CPUP-registry.

A cross-sectional study was performed using the most recent report from 2012 to 2014. Data were extracted for all children with CP born 2002–2013 and reported into the registry from January 1 2012 to June 30 2014. The prevalence of children with a confirmed CP diagnosis from the age of 4 years included in the registry in 2013 was 2.14/1000 (95 % CI 2.04–2.24). A number of studies including children and adults have been published using data from the CPUP registry [14], but not on this sub-set of children or the variables included in this study.

All assessments were performed by local occupational and physical therapists in a standardized manner according to the CPUP manual. The local therapists used the GMFCS levels I-V (expanded version) [4, 5]; MACS levels I-V [10]; House functional classification system of hand function based on activity levels ranging from 0 (does not use), to 8 (spontaneous use, manipulating hand) [17]; Zancolli [18] classification of spasticity of wrist/finger flexors grouped into 1–3, where 3 represents inability to actively extend fingers or wrist; thumb-in-palm deformities ranging from no deformity to type I-IV describing adduction contractures of metacarpal I with or without additional deformities of the metacarpophalangeal and interphalangeal joint [17]; and bimanual ability reported as ‘Yes’ or ‘No’ when assessing spontaneous use of each hand in activities requiring both hands. Passive joint range of motion of the upper limbs was measured with a goniometer using a protocol (http://cpup.se). If the CPUP neuro-paediatric consultation reported visual or cognitive impairment, these data were also extracted.

Information was collected on typical wheeled mobility performance (what the child did in everyday life, rather than what they might be capable of under ideal or test conditions). Parents reported whether their child used a wheelchair indoors and/or outdoors, and whether they were typically independent or needed adult assistance. All types of wheelchairs were included but strollers or buggies with four small wheels, designed solely for use by an attendant were not.

### Statistical analyses

The data were stratified into four groups according to the type of wheelchair (power, manual), and environment (indoors, outdoors). Children could be included in more than one group if they used manual and power, or used their wheelchair in both environments. The Cox Proportional Hazard models with an equal follow-up and robust variance were used. This approach allows interpretation of obtained hazard ratio as a prevalence ratio in cross-sectional studies [19, 20]. Hazard ratios (HR) with 95 % Confidence intervals (CI) and *p*-values were used, to calculate the risk for not being able to self-propel, for each analysed factor in a separate model that included one analysed factor adjusted for sex and age. In all analyses the worst values (between left and right side) were used. Significance level was set at *p* < 0.05. All analyses were performed using STATA 12 software.

Measures were grouped, based on whether a score was deemed to be a risk factor, limiting ability to operate a wheelchair and distribution of variables for analysis. For House functional classification system [17], active hand (House 4–6) and passive hand (House 0–3) were compared with the reference group manipulating hand (House 7–8). With Zancolli [18], groups 2A, 2B and 3 were grouped as less functional (more wrist and finger spasticity) and combined 1+X and 1 (more functional) as the reference category. Children were classified as ‘yes’ having thumb-in-palm deformity I-IV or ‘no’ with no thumb-in-palm as the reference category. Bimanual ability was scored as ‘yes’ or ‘no’ for active achievement. GMFCS levels [5] IV and V were compared with reference category levels I-III. MACS levels [10] could not be grouped in the same manner, as no children at MACS IV or V could self-propel a manual wheelchair. MACS IV and V were therefore combined and compared with MACS level III against the more able reference category of levels I-II.

Critical range of motion values set up by the CPUP programme were used to categorize the data. Green values signify almost full range of motion and were used as the reference category and analysed against the supposed risk factors yellow (warning) and red values (act) (http://cpup.se). Reference categories for range of motion were ≥160° shoulder flexion, ≥ − 10° elbow extension, ≥80° forearm supination, and ≥60° wrist extension.

## Results

Data from a total of 2328 children 0–11 years, 1344 (58 %) boys and 984 (42 %) girls were reported. The mean age of the children was 6 years 2 months (SD 2 years, 11 months). GMFCS and MACS levels are shown in Table 1 along with the percentages and numbers using manual and power wheelchairs. No child used a manual wheelchair before 12 months. For children aged between 1 and 2 years, 2 of 93 were pushed in a manual wheelchair indoors and 4 of 93 were pushed in a manual wheelchair outdoors. Between 2 and 3 years-of-age only 2 of 184 children used a manual wheelchair independently indoors. No child used a power wheelchair before age 3 years and children below 6 years were primarily considered to be independent in the power wheelchair only outdoors.

**Table 1:** Number and percentages (%) of children using manual and powered wheelchairs indoors and outdoors relative to the total number of children with cerebral palsy

		Manual Indoors		Powered Indoors		Manual Outdoors		Powered Outdoors		Total number of children
		Self-propels	Pushed	Drives	Assisted	Self-propels	Pushed	Drives	Assisted	
GMFCS	I	0	0	0	0	4 (0.5 %)	28 (3 %)	3 (0.5 %)	0	1016
	II	15 (4 %)	5 (1 %)	0	0	21 (6 %)	108 (30 %)	21 (6 %)	0	365
	III	64 (29 %)	21 (9 %)	10 (5 %)	0	27 (12 %)	127 (57 %)	35 (16 %	2 (1 %)	224
	IV	85 (24 %)	160 (45 %)	35 (10 %)	4 (1 %)	30 (8 %)	233 (65 %)	61 (17 %)	13 (4 %)	359
	V	1 (0.5 %)	248 (68 %)	2 (1 %)	20 (6 %)	1 (0.5 %)	259 (71 %)	5 (1 %)	26 (7 %)	364
MACS	I	25 (4 %)	3 (0.5 %)	1 (0 %)	0	31 (5 %)	49 (8 %)	16 (3 %)	0	645
	II	52 (11 %)	14 (3 %)	10 (2 %)	0	28 (6 %)	110 (23 %)	39 (8 %)	0	484
	III	48 (18 %)	37 (14 %)	24 (9 %)	1 (0.5 %)	13 (5 %)	119 (45 %)	37 (14 %)	4 (2 %)	266
	IV	29 (11 %)	123 (47 %)	10 (4 %)	5 (2 %)	5 (2 %)	190 (73 %)	26 (10 %)	10 (4 %)	261
	V	1 (0.5 %)	221 (71 %)	2 (0.5 %)	17 (6 %)	0	231 (75 %)	5 (2 %)	25 (8 %)	310
	UC	10 (3 %)	36 (10 %)	0	1 (0.5 %)	6 (2 %)	56 (16 %)	2 (0.5 %)	2 (0.5 %)	362
Age	<1	0	0	0	0	0	0	0	0	9
	1	0	2 (2 %)	0	0	0	4 (4 %)	0	0	93
	2	5 (3 %)	11 (6 %)	0	0	2 (1 %)	15 (8 %)	0	0	184
	3	13 (6 %)	24 (12 %)	1 (0.5 %)	1 (0.5 %)	2 (1 %)	44 (21 %)	1 (0.5 %)	2 (1 %)	207
	4	16 (7 %)	19 (8 %)	1 (0.5 %)	1 (0.5 %)	8 (3 %)	47 (20 %)	5 (2 %)	2 (1 %)	240
	5	19 (7 %)	48 (17 %)	1 (0.5 %)	1 (0.5 %)	10 (4 %)	93 (32 %)	10 (4 %)	4 (1 %)	287
	6	15 (6 %)	42 (17 %)	5 (2 %)	2 (1 %)	7 (3 %)	85 (34 %)	10 (4 %)	3 (1 %)	249
	7	16 (7 %)	50 (23 %)	4 (2 %)	5 (2 %)	8 (4 %)	89 (41 %)	21 (10 %)	7 (3 %)	216
	8	25 (11 %)	74 (32 %)	5 (2 %)	3 (1 %)	14 (6 %)	112 (48 %)	15 (7 %)	6 (3 %)	232
	9	17 (8 %)	51 (24 %)	5 (2 %)	0	10 (5 %)	84 (40 %)	17 (8 %)	2 (1 %)	215
	10	19 (9 %)	52 (23 %)	12 (5 %)	6 (3 %)	11 (5 %)	88 (40 %)	23 (10 %)	4 (2 %)	224
	11	20 (12 %)	55 (32 %)	13 (8 %)	10 (6 %)	11 (6 %)	90 (52 %)	23 (13 %)	14 (8 %)	172
Sex	Boy	105 (8 %)	246 (18 %)	25 (2 %)	15 (1 %)	55 (4 %)	445 (33 %)	73 (5 %)	28 (2 %)	1344
	Girl	60 (6 %)	188 (19 %)	22 (2 %)	9 (1 %)	28 (3 %)	310 (32 %)	52 (5 %)	13 (1 %)	984

*GMFCS* Gross motor function classification system, *MACS* Manual ability classification system

### Indoors

In total, 610 children (26 %) used wheelchairs indoors, 1715 did not and there were missing values for 3 children. Of the 610 children, 537 (88 %) used only manual, 11 (2 %) only power, and 62 (10 %) used both manual and power wheelchairs. Of the 599 children using manual wheelchairs, 165 (28 %) self-propelled and 434 (72 %) were pushed.

Only 71 children used power wheelchairs indoors; 47 (66 %) of these children drove independently while 24 (33 %) needed assistance. Of the 24 children aged 3 to 11 years who needed assistance with power mobility indoors, most (*n* = 20) were classified as GMFCS V and the remaining as level IV. Similarly, most (*n* = 17) were unable to handle objects (MACS V), five were MACS IV, one was MACS III, and one unclassified. Ten children had reports of severe visual impairments, 10 severe cognitive disabilities, and for 11 children data on co-existing impairments were not recorded.

### Outdoors

In total, 858 children (37 %) used wheelchairs outdoors, 1469 did not and there were missing values for one child. Of the 858 children, 692 (80 %) used only manual wheelchairs, 20 (2 %) only power, and 146 (17 %) used both outdoors. Only 83 (10 %) children self-propelled of the 838 who used manual wheelchairs and 755 (90 %) were pushed.

Of the 166 children using power mobility outdoors; 125 (75 %) drove independently and 41 (25 %) needed assistance. In the group of 41 children (3–11 years) reported to need assistance to drive outdoors, gross motor function was classified as GMFCS V (*n* = 26), GMFCS IV (*n* = 13) and GMFCS III (*n* = 2). The majority were classified as MACS V (*n* = 25) and the remaining children were classified as MACS IV (*n* = 10), MACS III (*n* = 4) and two were unclassified. Seventeen children had reports of severe visual impairments, 17 severe cognitive disabilities, and for 20 children data on co-existing impairments were not reported.

### Risk factors

Most children had good hand function and range of motion. Distributions of measurements for each category are presented in Table 2.

**Table 2:** Grouping of variables, number of children and missing values for each category

	Ref Cat	*N* of children	Cat 2	*N* of children	Cat 3	*N* of children	Missing
GMFCS	I-III	1605	IV	359	V	364	0
MACS	I-II	1129	III	266	IV-V	571	362
House	8-7	798	6-4	733	3-0	534	263
Zancolli	1X + 1	1366	2A, 2B, 3	308			654
Thumb-in-palm	No	1211	I-IV	836			281
Bimanual ability	No	440	Yes	1659			229
Shoulder flexion	≥160°	1927	<160° >120°	141	≤120°	25	235
Elbow extension	≥ −10°	2022	<−10° > −30°	51	≤ −30°	21	234
Forearm supination	≥80°	1907	<80° >45°	118	≤45°	70	233
Wrist extension	≥60°	1999	<60° ≥0°	77	<0°	7	245

*GMFCS* Gross motor function classification system, *MACS* Manual ability classification system, *Ref* Reference, *Cat* Category, *N* Number

Poor hand function (HR 2.4–5.4), spasticity of finger and wrist extensors (HR 2.2) and low level of gross motor function (HR 2.6–4) were all risk factors for not being able to self-propel manual wheelchairs, especially indoors (Table 3). The risk increased successively with lower levels of function. Upper extremity range of motion (HR 1.1–1.5) had some influence but not to the same extent as hand function. Limited shoulder flexion and wrist extension had more impact on manual mobility indoors than restricted supination or elbow extension.

**Table 3:**
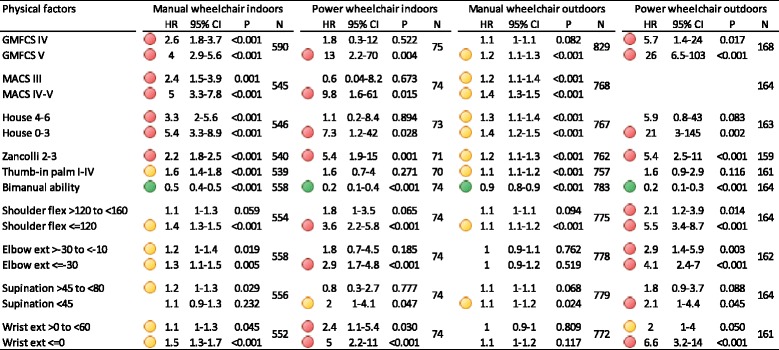
Hazard ratios (HR) with 95 % confidence intervals (CI) and *p*-values (P) adjusted for age and sex. Variables presented are treated as risk factors for not being able to self-propel. Number of observations included (*N*)

GMFCS I-III, MACS I-II, House 7–8, Zancolli 1, no thumb-in-palm, no bimanual ability and “green” values for range of motion were used as reference categories. Statistically significant (*p* < 0.05) Hazard ratios (HR) of 1.1–2 indicating an increased risk of 10–100 % are marked with yellow, HRs of >2 (more than doubled risk) are marked with red and HRs <1 (meaning a reduced risk is indicated with green)

The most important risk factors for not being able to operate a power wheelchair indoors were being classified as GMFCS V (HR 12.6) or MACS IV-V (HR 9.8). If the child did not have a manipulating hand or active grasp classified by House (HR 7.2), or had spasticity of the finger and wrist flexors rated as Zancolli 2–3 (HR 5.4), this reduced probability of independent power mobility, as did limited shoulder flexion (HR 3.6) and wrist extension (HR 5).

All risk factors had less influence on manual wheeled mobility outdoors with an increased risk of 0–40 % (HR 1–1.4) of not being able to self-propel. In comparison the risk of dependent power mobility was substantially higher for children with reduced hand function classified as House 0–3 (HR 20.7), spasticity in finger and wrist flexors (HR 5.4), limited shoulder and wrist range of motion (HR 5.5–6.6) and being classified as GMFCS V (HR 25.8).

## Discussion

This is the first study analysing the extent to which hand function, upper extremity range of motion, and gross motor function affect independent wheeled mobility performance, indoors and outdoors, in a total population of children with CP. One of four children with CP up to 11 years-of-age used a wheelchair for mobility indoors. Manual wheelchairs were only self-propelled by 28 % of the children indoors and by 10 % outdoors, while a vast majority were passively pushed by another person. More children used wheelchairs outdoors than indoors.

The majority of children did not self-propel their manual wheelchairs, particularly outdoors, regardless of age, gross motor function, upper extremity range of motion or manual abilities. In comparison, relatively small numbers of children used power mobility but the majority of these were able to achieve independence. Outdoors power wheelchairs were operated independently by 75 % of the 166 children while 25 % needed assistance, in some cases related to severe visual, cognitive or motor impairments.

These results provide support for previous studies, showing that power wheelchairs provide independent mobility while manual wheelchairs only facilitate care [21]. Levels of independence in manual mobility were similar in a previous study including children with CP [6] aged 3 to18 years with 86 % (189/219) being pushed outdoors in comparison with 90 % (755/838) in this study of 0–11 year-olds. Independent use of power mobility outdoors was slightly less in this study with 75 % (125/166) children driving independently in comparison with 86 % (48/56) reported for 3–18 year-olds. This may reflect the difference in age-span with expectation of independent mobility being lower for children under 3 years.

Independent manual mobility indoors was mostly affected by hand function (House classification), MACS and GMFCS levels. Limited upper extremity range of motion had a greater impact on manual mobility indoors than outdoors. This was unexpected as it might reasonably be anticipated that more upper extremity range of motion would be needed when wheeling outdoors. For manual mobility outdoors, more impaired MACS level was the highest risk factor. No active grasp [17], wrist and finger spasticity [18] and more impaired GMFCS level all increased the risk slightly but less than anticipated. Factors other than gross and fine motor function such as limited physical endurance, respiratory function, other personal or environmental factors may be influential but were not examined in this study. In addition, limited data were available on potentially influential co-existing impairments such as cognitive or visual limitations.

One striking result is that 36 % of children classified as GMFCS II (and therefore able to ambulate without aids indoors and outdoors) are not only using manual wheelchairs outdoors, but only 6 % can do so independently while 30 % are passively pushed. It appears that children with CP up to 11 years-of-age (at all GMFCS and MACS levels) are unlikely to attain independent mobility in manual wheelchairs outdoors irrespective of hand function, upper extremity range of motion or gross motor function abilities. So, manual wheeled mobility is not a realistic goal for the majority of children with CP. This strongly suggests that power mobility and other mobility options must be considered earlier for children at GMFCS levels II-V to promote independent mobility, activity and participation in the home, school and community.

For independent power mobility indoors, the highest risk factors were poor gross motor function (GMFCS V) and more impaired hand function, followed by wrist and finger spasticity. These factors also predicted a higher risk for dependent power mobility outdoors along with decreased upper limb range of motion. Children with limited purposeful hand use, including those classified as MACS and GMFCS V, may achieve independence using alternate access methods such as switches or head array for power mobility [2, 4, 22]. The fact that hand use and upper limb range of motion appeared to have such an impact on independent power mobility may suggest that most children were using standard side-mounted joysticks and alternate access methods may not have been adequately explored. However, information on power mobility access method is not collected in the CPUP database and so these data could not be analysed.

Environmental barriers may also have influenced study results. Children were included from all regions of Sweden including urban and rural areas. While environmental factors other than indoor and outdoor wheeled mobility performance were not specifically analysed, the data represents children living in the full range of cold northern to southern temperate weather regions, and both urban and rural environments. The wheeled mobility use data are therefore representative of the total population of children with CP in Sweden and suggests that results may be extrapolated to other countries that include a similar range of environments.

The range of outdoor environments included in this study may have influenced outdoor use of both manual and power wheelchairs. The outdoor environment is more challenging, and less familiar. There are longer distances to travel, more complex terrain (curbs, gravel, potholes), challenges when using public transports, different weather conditions (snow, ice, rain), and other potential hazards such as traffic to be negotiated. Outdoor terrain may be more challenging for younger children as well as those with visual impairments or cognitive limitations. Expectations should be age-appropriate as we would not expect young children who are walking to negotiate traffic or other hazards safely without adult assistance.

Surprisingly low numbers of power wheelchairs were prescribed for children with CP in this study. No child under the age of three years, and only 19 % of the total number of children using wheelchairs outdoors were prescribed power wheelchairs. Recent expert consensus confirms that children classified as GMFCS V will never be able to walk and should be considered for power mobility starting around 12 months of age [2]. According to our data 30 % of children classified as GMFCS II, 57 % as level III and 65 % as level IV were pushed in manual wheelchairs outdoors. These children who may be able to walk with or without aids but are unable to keep up or participate with their peers have inefficient mobility. They often benefit from power mobility to enhance activity and participation at school, outdoors, and in the community [1–3, 23].

In Sweden, wheelchairs are provided on loan to children/families without charge and can be replaced at any time as needs change. So reasons for this lack of prescription are not financial but may include other factors. In a cold climate there are difficulties managing power wheelchairs due to the need for a heated space for storage, and use outdoors in snow can be challenging. The increased size and weight of power wheelchairs can also lead to increased transportation difficulties. Attitudinal and social factors may relate to lack of ‘readiness’ of parents to accept a power wheelchair [11], and therapists may reinforce the perception that power mobility is only considered after other mobility methods have proved ineffective. A recent synthesis of the qualitative literature suggests that multiple factors in the physical, attitudinal and social environment influence power mobility access and use [1].

When motor development was seen as hierarchical, children were expected to walk as much as possible [24]; manual wheelchairs were provided when walking had failed and power wheelchairs were provided only when manual wheelchair use had failed. In contrast, current motor theories and research emphasise addressing constraints in the task and environment in order to improve function, activity and participation. The results of this study highlight that there is still a significant gap between contemporary theory and evidence and clinical practice.

In Sweden, in contrast to some other parts of Europe but similarly to North America, children with CP are integrated into mainstream childcare centres, preschools and school settings. This can lead to concerns about children’s safety (and their peers) especially given that power wheelchairs in Sweden are recycled and often large, powerful adult sized bases are used with small paediatric seats. Children aged 7–16 years have discussed their fears of hurting themselves or others, as well as the challenges of using large and powerful bases in less than accessible environments [25]. This further reinforces the need for smaller, more manoeuvrable power devices appropriate for use in early childhood settings [26].

There are a number of limitations to this study. The CPUP database lacks detailed information on the wheelchair type or access method (e.g. joystick, switches, head array) and information on postural control, strength or endurance. Data were incomplete for cognitive and visual abilities and these factors may have clarified some unexpected results. There were missing data for some variables that reduced the numbers included in the statistical analyses. Postural stability, often a problem for children with CP [27], may have significant impact on ability to self-propel. For many children with spastic diplegia, using hands and arms for manual wheeling often causes overflow tone that increases postural instability and increases difficulties with upper extremity use. Details of equipment factors may have been helpful in clarifying which children with higher risk factors are successful with power mobility or why upper limb range of motion appears to have more of an impact on power mobility outdoors. In addition, information on the specific activities and environments where children were using wheeled mobility devices as well as family perceptions and attitudes may have been helpful but was not available for analysis. It was also beyond the scope of this study to ascertain the reasons why power mobility was not more frequently prescribed however, anecdotal evidence suggests clinicians and or parents may continue to harbour negative attitudes towards introducing it, especially at younger ages.

A strength of this study is that it includes a total population of children with CP and is the largest study published to date including children at all GMFCS, MACS and cognitive levels analysing physical factors influencing use of manual and power wheelchairs indoors and outdoors. The numbers of children shown as being dependent and independent corresponds to previous data reported from studies in Canada [7] and Sweden [6] and so are likely representative for children with CP in similar environments.

## Conclusions

A vast majority of children with CP, aged 0–11 years did not self-propel manual wheelchairs regardless of age, gross motor function, range of motion or manual abilities. Power mobility should be considered at earlier ages to promote independent mobility for children with CP who require a wheelchair especially outdoors. With all available research on the benefits of enhancing early mobility and the growing evidence that manual wheelchairs are not the solution for children with CP who wish to attain independent wheeled mobility regardless of age and functional level, it is concerning that power mobility was not prescribed for greater numbers of children or at younger ages. Sadly, the results are similar for older children and adolescents [6], so we have to move from theory to practice and explore all mobility options to promote independence, activity and participation for children with CP. There is need for further research to help identify other factors influencing independence in efficient wheeled mobility for children. This should include further exploration of the interaction between factors in the child and the environment. This may assist clinicians in enabling children to effectively participate in all settings.

## Abbreviations

CP: Cerebral palsy
CPUP: Swedish cerebral palsy surveillance programme and National quality registry
GMFCS: Gross motor function classification system
HR: Hazard ratio
MACS: Manual ability classification system
